# Dose enhancement and cytotoxicity of gold nanoparticles in colon cancer cells when irradiated with kilo‐ and mega‐voltage radiation

**DOI:** 10.1002/btm2.10007

**Published:** 2016-05-27

**Authors:** Herman Hau, Dipesh Khanal, Linda Rogers, Natalka Suchowerska, Rajiv Kumar, Srinivas Sridhar, David McKenzie, Wojciech Chrzanowski

**Affiliations:** ^1^ Faculty of Pharmacy The University of Sydney New South Wales 2006 Sydney, Australia; ^2^ Chris O'Brien Lifehouse Sydney New South Wales 2006 Sydney, Australia; ^3^ School of Physics The University of Sydney New South Wales 2006 Sydney, Australia; ^4^ Nanomedicine Science and Technology Center and Dept. of Physics Northeastern University Boston MA 02115; ^5^ Charles Perkins Centre, The University of Sydney New South Wales 2006 Sydney, Australia; ^6^ Australian Institute of Nanoscale Science and Technology The University of Sydney, New South Wales 2006 Sydney, Australia

**Keywords:** colon cancer, gold nanoparticles, magnetic levitation, radiation dose enhancement, three‐dimensional cell culture

## Abstract

Despite major advances in the field of radiotherapy, healthy tissue damage continues to constrain the dose that can be prescribed in cancer therapy. Gold nanoparticles (GNPs) have been proposed as a solution to minimize radiation‐associated toxicities by enhancing the radiation dose delivered locally to tumor cells. In the current study, we investigated the application of third‐generation GNPs in two‐dimensional (2D) and three‐dimensional (3D) cell cultures and whether there is synergy between the nanoparticles and kilo‐ or mega‐voltage radiation to cause augmented cytotoxicity. The 10‐nm GNPs were found to be nontoxic in both 2D and 3D in vitro cultures of colon cancer cells at concentrations of up to 10–25 µg/ml. There was a significant increase in cell survival fraction reduction following exposure to 1 Gy of kilo‐voltage (18.3%) and 2 Gy of mega‐voltage (35.3%) radiation when the cells were incubated with 50 µg/ml of GNPs. The biocompatibility of the GNPs combined with their substantial synergy with radiation encourages further investigations into their application in targeted cancer treatment.

## 1 Introduction

According to the World Health Organization, cancer is a leading cause of death globally.^1^ Ionizing radiation has been utilized as a form of cancer treatment in radiotherapy since the late 19th century.^2^ Radiotherapy, however, is not free from side effects, which arise from the interactions of the radiation beam with healthy tissues, leading to tissue damage and/or scarring. Targeted delivery of x‐rays has been refined over time to minimize skin and healthy tissue damage^3^ and also allow any site in the body be irradiated with millimeter positional accuracy and centiGray dose precision. A logical approach would be to exploit synergies between existing modes of treatment to reduce the side effects of each individually and enhance treatment efficacy. In recent decades, the use of nanoparticles with radiotherapy shows considerable promise.

Gold nanoparticles (GNPs) are of interest as a method for improving radiotherapy.^4^ The aim of using nanoparticles in radiotherapy is to selectively induce higher toxicity to tumors than to normal tissue.^5^ Specific research interest in GNPs arises from their preferential accumulation in tumors through the enhanced permeability and retention effect, their high surface area to volume ratio as well as their ease of functionalization through thiol functional groups.^4^, ^6^, ^7^, ^8^

Research into GNPs in radiotherapy has long demonstrated that the enhancement of radiation dose to cancer cells is possible.^9^ However, there are still numerous questions regarding GNPs that have yet to be answered. First, the biocompatibility of GNPs continues to be questioned. There are publications that suggest that toxicity is dependent on size,^10^, ^11^ shape,^12^ surface chemical properties,^13^ the particular cell type and model being tested.^14^ With so many variables and no common preparation or procedure with which GNPs toxicity can be investigated, the direct comparison of research results is extremely challenging, if not impossible.

Second, the radio‐enhancement effect of GNPs may also be dependent on size,^15^ energy of radiation,^16^ and cell type.^17^ The interplay of cell line and radiation energy on radio‐enhancement is well illustrated by Jain et al.^16^ A statistically significant (40%) reduction in the mean inactivation radiation dose was achieved in MDA‐MB‐231 breast cancer cells using 160 kVp x‐rays. However, no significant reduction was observed in DU145 human prostate cancer cells or L132 lung epithelial cells despite confirmation of GNP uptake. Moreover, increasing the energy of the radiation to 6 MV led to 29% reduction in mean inactivation dose in MDA‐MB‐231 cells but in DU145 cells, only a 13% reduction was observed. This shows that even identical experimental protocols by the same research group yields results that display no obvious trend or tendencies.

Despite these difficulties, GNPs demonstrate great potential in radiotherapy and cancer treatment. Further research and innovations in this field will enable the eventual establishment of the true effectiveness of GNPs in radiotherapy.

Here, we report the results of an investigation into the biocompatibility as well as the synergy between GNPs and ionising radiation. We demonstrated the potential of GNPs to increase cell death. Specifically, we evaluated the in vitro toxicity of our GNP formulation in LOVO human colon cancer cells in both a two‐dimensional (2D) culture and three‐dimensional (3D) model. The radiation enhancement and synergistic effects with both 50‐kVp x‐ray and 6‐MV photon radiation were determined using the clonogenic assay.

## 2 Materials and Method

### 2.1 GNPs synthesis

The GNPs were synthesized by reduction of auric chloride using a method previously outlined.^18^ In brief, tetrakis (hydroxymethyl) phosphonium chloride (80%) was added into an alkaline solution while stirring, followed by the addition of gold (III) chloride trihydrate. This turns the mixture from a yellow to a dark brown color, indicating the formation of the GNPs. The GNPs were then used as is as non‐polyethylene glycol (PEG)ylated GNPs (nGNP) or were further PEGylated with three different functionalized polyethylene glycols [m‐PEG‐thiol (MW 2,000), carboxymethyl‐PEG‐thiol (MW 2,000), and amine‐PEG‐thiol (MW 3,400)] for stabilization and future conjugation of substrates (PEGylated GNP [pGNP]). The unreacted PEGs were removed by dialysis using 14k MW cutoff cellulose membrane tubing. The resultant solution was then use as a solution or freeze dried to obtain dry powder‐like GNPs.

### 2.2 GNPs characterization

#### 2.2.1 Electrophoretic light scattering

The surface charge (ζ‐potential) of the nGNPs and the pGNPs were determined using a Zetasizer Nano ZS (Malvern Instruments, Worcestershire, U.K.). Samples were tested at room temperature (25°C) with a refractive index of 1.

#### 2.2.2 Dynamic light scattering

The size of the nGNPs and the pGNPs were determined using a Zetasizer Nano ZS (Malvern Instruments). Samples were tested at room temperature (25°C) with a refractive index of 1. The mass median diameter was reported.

#### 2.2.3 Atomic force microscopy

Atomic force microscopy (AFM) on nGNPs and pGNPs were conducted in tapping mode using a NanoIR (Anasys Instruments, CA). The particles were fixed onto a mica disk by poly‐lysine in order to be imaged.

#### 2.2.4 Scanning transmission electron microscopy

Scanning transmission electron microscopy (STEM) was performed using a Carl Zeiss Ultra Plus Field Emission Gun SEM. Copper 100 mesh STEM grids with formvar film (Proscitech, Australia) were dipped into a small drop of each GNP solution. The grid was quickly retracted and allowed to dry. The images were taken at an acceleration voltage of 30 kV using a STEM detector.

### 2.3 In vitro investigations

#### 2.3.1 Live/Dead assay

The viability of cells treated with pGNPs was investigated using the Live/Dead assay. The assay was carried out as per the manufacturer's protocol. Briefly, LOVO cells (6 × 10^3^) were seeded into 96‐well plates and were allowed to attach for 4 hr. Culture medium was replaced with fresh medium containing different concentration of pGNPs (10, 25, and 50 µg/ml) and incubated for 7 days. Cells were washed with phosphate buffered saline (PBS) and treated with 2 μM Calcein AM and 4 μM EthD‐III in PBS and incubated in the dark for 45 min at 37°C. The number of live (green) and dead (red) cells were evaluated qualitatively based on the fluorescent images (Nikon Eclipse TE2000‐U inverted fluorescent microscope, Melville, NY, USA).

#### 2.3.2 Live cell imaging

Effect of pGNPs on cell proliferation was investigated by recording time lapse images of the cells over 7 days. LOVO cells (6 × 10^3^) were seeded into 96‐well plates and allowed to attach for 4 hr. The media was aspirated and replaced with fresh medium containing different concentrations of pGNP (10, 25, and 50 µg/ml). The plate was imaged in phase using an IncuCyte ZOOM™ Kinetic Imaging System (Essen Bioscience) every 1 hr for 7 days. Inbuilt software was used for analysis of the images to generate confluence data.

#### 2.3.3 3D cell migration assay

The ring closure assay was carried out as per the protocol previously reported in the literature.^19^ Briefly, LOVO cells incubated with Nanoshuttle^®^ were levitated with neodymium magnets overnight to form aggregated 3D clusters, which were then dispersed using a pipette. A cell density of 2 × 10^5^ cells per well was seeded to an ultralow attachment 96‐well plate placed on top of a 96‐well ring‐shaped magnetic drive. Cells were incubated for 1 hr to form a robust 3D ring structure. pGNPs (10, 25, 50, and 100 µg/ml) were added to respective wells with *n* = 3 per concentration. The cell rings were monitored using a mobile device (Ipod Touch, Apple computer) with a specific application (Experimental assistant, n3D Biosciences) that takes images at 15 min intervals. The images were analyzed using a custom n3D software image analysis code written in MATLAB (Matworks, Natick, MA) to measure the outer diameters of the ring. The percentage change in diameter was assessed by normalizing the diameters to its initial diameter. Phase images of the cell rings at each concentration were taken after 24 hr using a Nikon Eclipse TE2000‐U inverted fluorescent microscope.

#### 2.3.4 3D spheroid assay

The assay was carried out as previously outline.^20^ Briefly, the Nanoshuttle^®^ treated LOVO cells were levitated with neodymium magnets overnight to form aggregated 3D clusters. A cell density of 1 × 10^5^ cells per well was seeded to an ultralow attachment 96‐well plate placed on top of a 96‐well dot‐shaped magnetic drive and proceeded as per the migration assay. The phase images of the spheroids after 24 hr at each concentration were captured using the JuLi Stage (NanoEnTek, Seoul, Korea).

### 2.4 GNP potentiation of radiation dose

#### 2.4.1 Cell culture

LOVO cells were obtained from American Type Culture Collection (ATCC) and were maintained in Dulbecco's modified eagle's medium (Gibco Life Technologies, Australia) supplemented with 10% vol/vol foetal bovine serum (Gibco Life Technologies) as recommended by ATCC and maintained in a humidified incubator with 5% CO_2_ at 37°C. To minimize stress on the cells, no antibiotics or antifungal agents were used in these experiments.^21^ Once 80% confluent, the growth medium was discarded and the adherent cells were washed with PBS (Gibco Life Technologies) twice. Cells were then detached using .05% trypsin‐EDTA solution for 4–6 min at 37°C as previously described.^22^ The detached cells were centrifuged at 252 × *g* for 5 min at room temperature. The cells were then plated in T25 cm^2^ flasks (Corning, MA) at a density of 1,500 cells in 5‐ml growth medium.

#### 2.4.2 Kilo‐voltage radiation dose determination

Twenty four hours after plating, each flask was supplemented with 20 ml of media. Irradiation was carried out a further 24 hr later on a Pantak Orthovoltage Unit using a 50‐kVp beam to a uniform dose of 1 Gy. For all experiments, unexposed controls (0 Gy) were prepared as sham exposures.

#### 2.4.3 Mega‐voltage radiation dose determination

Twenty four hours after plating, each flask was supplemented with 20 ml of media. Irradiation was carried out a further 24 hr later on a Varian Novalis TX linear accelerator with a 6‐MV photon beam at a dose rate of 6 Gy/min. To achieve full scatter conditions, flasks containing the cells were placed in a custom built Perspex phantom that accommodates T25 flasks. The phantom was placed between slabs of solid water to locate the cell layer at a depth of 50 mm when irradiated at gantry 180° as previously described.^23^ The cells were irradiated to a uniform dose of 2 Gy. For all experiments, unexposed controls (0 Gy) were prepared as sham exposures.

#### 2.4.4 GNP concentration determination

Twenty four hours after plating, each flask was supplemented with 20 ml of media modified with different concentrations of pGNPs (2, 4, 5, 10, 25, and 50 µg/ml) and further incubated for 24 hr.

#### 2.4.5 Investigation of synergistic effect of GNPs and radiation by clonogenic assay

Twenty four hours after plating, the media was replaced with 20 ml of new media or new media modified with addition of 50 µg/ml of pGNPs for the treated culture and incubated for 24 hr. The cells were then irradiated in air with either 2 Gy of 6‐MV x‐ray or 1 Gy of 50‐kVp x‐ray as outlined above.

#### 2.4.6 Clonogenic survival assay

Following the above treatment/s, all flasks were incubated at 37°C for 10 days until colonies of greater than 50 cells formed, then were fixed and stained with .4% crystal violet (Sigma‐Aldrich, MO). The number of colonies were counted using ColCount colony counter (Oxford Optronix, U.K.). All experiments were carried out in triplicate and on three separate occasions.

### 2.5 Statistics

Statistically significant differences were determined with Prism 6 (GraphPad Softrware, CA) by one‐tailed unpaired *t* test or one‐way analysis of variance (ANOVA) with a *p* value of .05 or less considered significant.

## 3 Results

### 3.1 GNP characterization

Both the nGNP and pGNP were tested using electrophoretic light scattering to determine their ζ‐potential. The size measurements of nGNP and pGNPs were carried out using dynamic light scattering, AFM, and STEM.

The ζ‐potential and the size of the GNPs increased by 27.9 mV and 3.3 nm, respectively, after the PEGylation process (Table 1).

**Table 1:** ζ‐Potential and mass median diameter of non‐PEGylated and PEGylated GNPs

Particle type	ζ‐Potential (mV)	Size (nm)
Non‐PEGylated gold nanoparticles	−33.3	7.5
PEGylated gold nanoparticles	−5.4	10.8

**Figure 1:**
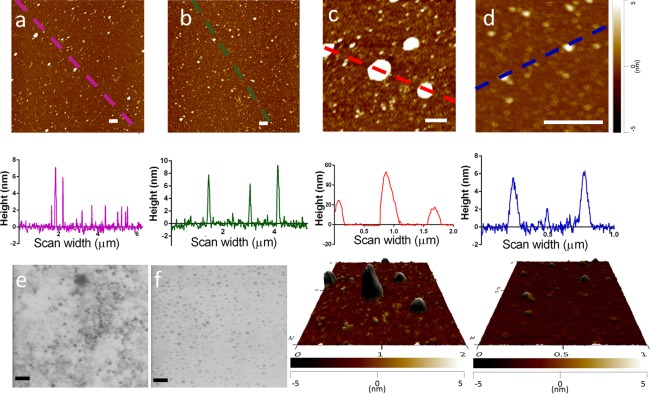
Gold nanoparticles sizing by AFM and STEM. (a) Large‐scale topographic AFM image of the nGNPs fixed to mica, with a corresponding profile analysis below (scale bar = .5 µm). (b) Large‐scale topographic AFM image of the pGNP fixed to mica, with its corresponding profile analysis below (scale bar = .5 µm). (c) Small‐scale topographic AFM image of the nGNPs fixed to mica, with a corresponding profile analysis and 3D representation below (scale bar = .5 µm). (d) Small‐scale topographic AFM image of the pGNPs fixed to mica, with a corresponding profile analysis and 3D representation below (scale bar = .5 µm). (e) STEM image of the nGNPs (scale bar = 20 nm). (f) STEM image of the pGNPs (scale bar = 20 nm)

Figure 1a shows the large‐scale AFM image taken of nGNPs with the corresponding line profile analysis below. The profile analysis demonstrated that the size of the individual nGNPs was in the range of 3–7 nm. A smaller scale AFM image of the same sample with its corresponding profile analysis and 3D representation (Figure 1c) indicates the presence of additional larger structures. These aggregates varied in size from approximately 20–45 nm. The large‐scale AFM image of the pGNPs and its profile analysis (Figure 1b) shows a general increase in size, resulting in sizes ranging from 5 to 9 nm. The larger structures observed in the nGNP samples were absent from the pGNP sample (Figure 1d).

While individual nGNP can still be discerned under STEM (Figure 1e), the particles appear to cluster together. In comparison, the pGNPs are dispersed uniformly (Figure 1f), with a clear difference in the pattern of distribution from nGNPs. In both cases, the particles appear to be under or equal to 10 nm.

### 3.2 In vitro toxicity and viability

**Figure 2:**
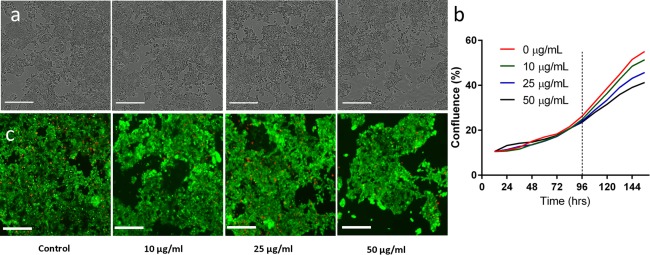
Evaluation of pGNP toxicity in LOVO cells in 2D culture by cell growth and viability. (a) Phase images of LOVO cells treated with 0, 10, 25, and 50 µg/ml of pGNP for 7 days (scale bar = 300 µm). (b) Quantitative analysis of the confluence of LOVO cells treated with 0, 10, 25, and 50 µg/ml of pGNP over 7 days. (c) Live/Dead assay of LOVO cells treated with 0, 10, 25, and 50 µg/ml of pGNP over 7 days (scale bar = 300 µm)

LOVO cells were exposed to different concentrations of GNPs for 7 days and their proliferation and viability were measured using live cell imaging and Live/Dead assay, with results as shown in Figure 2a. Analysis of the phase contrast images suggests a decline in cell proliferation at higher concentrations, which is exemplified by the quantitative measurement of the cell confluence (Figure 2b). The Live/Dead assay results suggest that the cells remain viable despite exposure to higher concentrations (50 µg/ml) of pGNPs.

**Figure 3:**
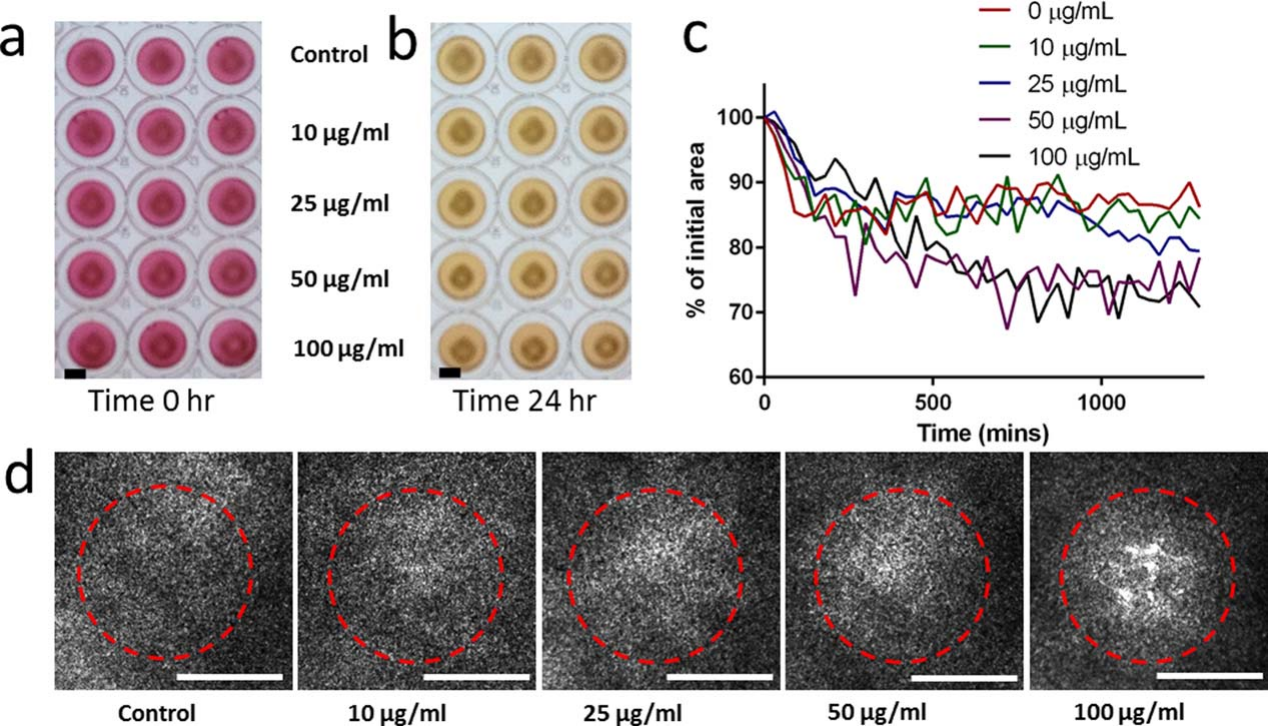
Evaluation of pGNP toxicity in LOVO cells cultured in 3D ring model. (a) Photograph of LOVO cell rings incubated with 0–100 µg/ml of pGNP at time = 0 hr (scale bar = 5 mm). (b) Photograph of LOVO cell rings incubated with 0–100 µg/ml of pGNP at time = 24 hr. (c) The contraction of the ring area over time as a percentage of its initial ring area. (d) Phase contrast images of the centre of LOVO cell rings after 24‐hr incubation with 0–100 µg/ml of pGNP (scale bar = 500 µm). Red dotted lines have been included to highlight the difference in extent of migration

LOVO cells were magnetically manipulated to form 3D ring structures and exposed to different concentrations of pGNPs over 24 hr. The cell's migration toward the center of the ring and growth measured by expansion of the outer ring diameter was monitored over time to assess toxicity. Changes to the cell rings after 24 hr were photographed macroscopically (Figures 3a and 3b). Quantitative analysis of changes to the averaged size of the external diameter of the rings over time at different concentrations of pGNPs (Figure 3c) demonstrates that there is a statistically significant (One‐way ANOVA multiple comparisons) difference between the control and the 50 µg/ml (*p* < .0001), and the 100 µg/ml samples (*p* < .0001). The migration of the cells toward the center of the ring was only examined qualitatively due to limitations of the analysis software. However, we can see a very slight difference in migration between different concentrations of pGNPs, with only small gaps (incomplete ring closure) visible at 100 µg/ml (Figure 3d). Red dotted rings were included in Figure 3d to highlight the difference in the extent of migration.

**Figure 4:**
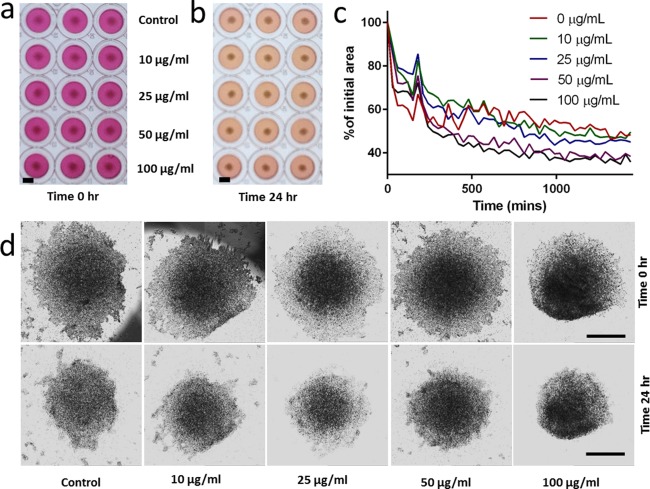
Evaluation of pGNP toxicity in LOVO cells cultured as 3D spheroids. (a) Photograph of LOVO cell spheroids incubated with 0–100 µg/ml of pGNP at time = 0 hr (scale bar = 5 mm). (b) Photograph of LOVO cell spheroids incubated with 0–100 µg/ml of pGNP at time = 24 hr. (c) The contraction of the spheroid's area over time as a percentage of its initial area. (d) Phase contrast images of the LOVO cell spheroids at time = 0 and after 24‐hr incubation with 0–100 µg/ml of pGNP (scale bar = 500 µm)

To analyze further the effect of pGNPs on cells, LOVO cells were also magnetically manipulated to form 3D spheroid structures and exposed to different concentrations of pGNPs over 24 hr. The change in area of the spheroid was monitored to assess cell toxicity. Changes to spheroids were apparent over the 24‐hr period (Figures 4a and 4b). As per the ring assay, quantitative analysis indicates a statistically significant (One‐way ANOVA multiple comparisons) difference in the reduction of the spheroid size at 50 µg/ml (*p* < .0045) and 100 µg/ml (*p* < .0001) when compared to control (Figure 4c). The phase images of the spheroids before and after 24‐hr incubation are shown in Figure 4d.

### 3.3 GNP potentiation of radiation dose

**Figure 5:**
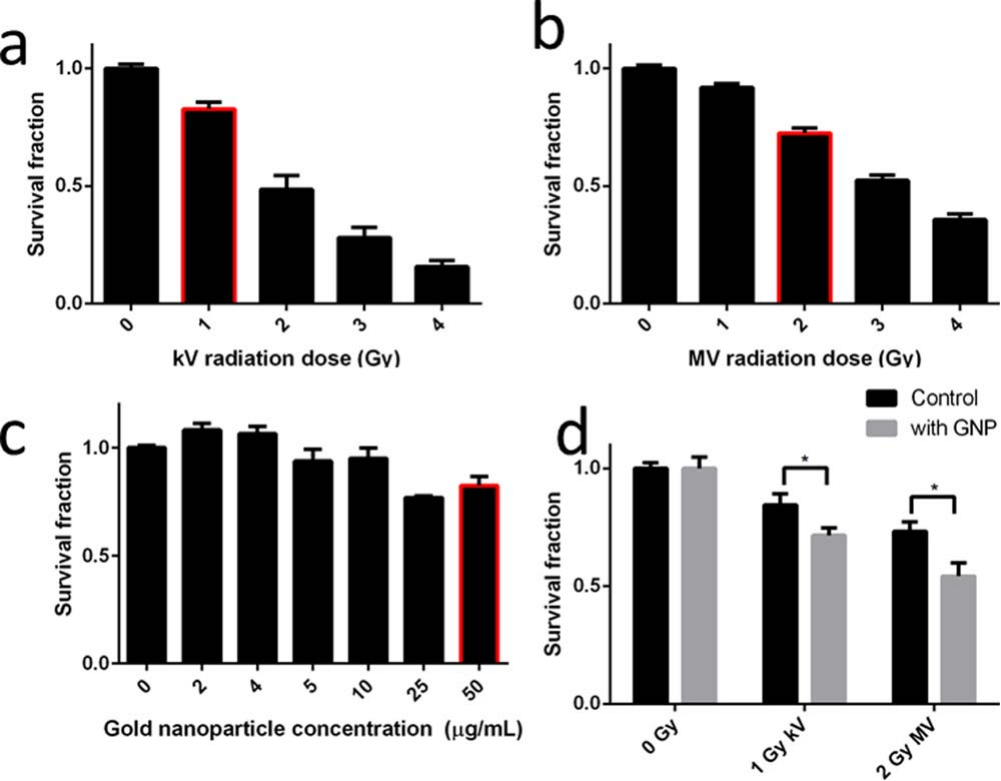
Result of dose determination of (a) kilo‐voltage radiation, with the red highlighted dose being chosen for the study, (b) mega‐voltage radiation, with the red highlighted dose being chosen for the study, (c) GNPs concentration, with the red highlighted dose being chosen for the study. (d) The survival fraction of LOVO cells with or without incubation with 50 µg/ml of GNP and treated with kilo‐voltage or mega‐voltage radiation

The LOVO cell survival fractions at different doses of kilo‐ and mega‐voltage radiation, as well as different concentrations of pGNPs are shown in Figures 5a–5c, respectively. The results of differences in survival fractions of LOVO cells with or without 50 µg/ml pGNPs at different radiation energies are shown in Figure 5d. To account for the toxicity of the pGNPs, the survival fractions of the samples incubated with pGNPs has been normalized to its corresponding control. Post‐normalization, there is a statistically significant (one‐tailed equal variance *t* test) difference between the survival fraction of cells when incubated with and without pGNPs in the case of both kilo‐voltage (*p* < .03) and mega‐voltage (*p* < .05). For kilo‐voltage irradiation, the survival fraction without pGNPs declines from.84 to .71 with pGNPs, which equates to an 18.3% further reduction. Similarly, the survival fraction of mega‐voltage irradiated cells decreases from .73 without pGNPs to .54 with pGNPs, representing a 35.2% increase in cytotoxicity.

## 4 Discussion

The change in ζ‐potential after the PEGylation process confirms that the PEG molecules are grafted on to the GNPs surface. This is further confirmed by the increase in size of the GNPs after PEGylation, as determined by electrophoretic light scattering. AFM images also revealed an overall increase in the particle size. Combined, both techniques along with STEM suggest that the particles were approximately 10 nm after PEGylation. A particle size of 10 nm was considered favorable, as we hypothesize that smaller GNPs would maximize the potential of secondary electrons to escape the nanoparticles, thereby maximizing the damage to tumor cells. Also, while compared to particles of 1–2 nm, 15‐nm GNPs have been found to be comparatively nontoxic.^10^ Therefore, 10‐nm GNPs were proposed to be the optimal size and were used in further experiments.

The importance of PEG in stabilizing the GNP is highlighted by AFM and STEM. The small‐scale AFM images of the nGNP and pGNPs and their respective profile analyses below (Figures 1c and 1d) indicate that there is considerable size difference between them. The nGNP sample shows structures up to 45 nm in size, which were absent from the pGNP sample. These large structures were attributed to the aggregation of the nGNPs, which aggregate to varying extents to form particles ranging from 20 to 45 nm. This stabilization effect is also demonstrated in the STEM images. The even dispersion and clear separation of individual pGNPs is in high contrast to the clustered nGNPs.

The phase contrast images of cell confluence and its quantitative analysis indicate that there is a dose‐dependent decrease in proliferation of the cells when exposed to pGNPs. It should be noted that the difference in proliferation rate begins to occur only after 96 hr of incubation and appears to continually diverge further as incubation time increases. This finding is of interest as many toxicity studies in the literature incubate the cells with GNPs for only 24–72 hr.^10^, ^16^, ^24^, ^25^, ^26^ This suggests that the toxicity profile of GNPs can change over time at different concentrations. We speculate that this is due to increasing uptake of GNPs as exposure time increases. It has been reported that both 5‐ and 15‐nm GNPs continue to be taken up by Balb/3T3 mouse fibroblasts for up to at least 72 hr.^27^ In addition, the accumulation of GNPs in cell lysosomes has been found to impair lysosome degradation capacity, ultimately affecting cellular homeostasis.^28^ Therefore, we propose that increased uptake of GNPs by prolonged incubation time has a cumulative negative effect on cellular homeostasis, leading to retarded cell proliferation at higher concentrations over time.

The viability of the cells does not seem to change with increasing pGNPs as suggested by the Live/Dead assay. However, as in the phase contrast image there is an obvious decrease in the confluence of the cells, especially at 50 µg/ml. Given that we see a decrease in proliferation but no change in viability, it may be possible that the dead cells detached during the washing process of the assay. This would lead to healthy green cells being apparent in the assay, while the absolute number of cells decreases. Alternatively, the cells may sense that there is a “toxin” in its environment, which leads to slower proliferation and migration toward other cells to form tight clusters to minimize exposed surface area and toxicity.

The growth of cells was also evaluated using 3D cell models, which more accurately portrays the actual biochemical environment of the cells in vivo than traditional 2D cell culture.^19^ The toxicity of specific compounds has been found to be different in the two culture types, with 3D models often being more resilient than 2D models.^19^, ^29^ Therefore, for the current study, the maximum pGNP concentration was increased from 50 to 100 µg/ml to assess the difference, if any, of the two culture types in their response to pGNPs.

In the 3D ring closure and spheroid assay, the general trend of ring size reduction for 25 µg/ml follows more closely to that of the lower 10 µg/ml as oppose to the 50 µg/ml observed in the 2D culture (Figures 3c and 4c). This supports the trend that cells in 3D cultures tends to be more resilient than in 2D culture.

The contraction of the ring size was significantly higher at both 50 and 100 µg/ml when compared to the lower concentrations. This was a surprising result as it was previously stated that toxic compounds should cause the spheroid to contract at a slow rate.^20^ Variability in response to toxicity has been found to arise from differences in the extracellular matrix composition as well as cell to extracellular matrix interactions.^19^ Therefore, it is possible that the LOVO cells actually contract at a higher rate in response to toxicity, which corresponds exactly to their behavior of contraction/clustering to form smaller colonies in the 2D culture. This argument is further supported by the closing of the internal gap of the ring (Figure 3d), which is akin to a 3D wound healing assay.^19^ At up to 50 µg/ml, the cells were able to migrate quickly to completely close the gap of the ring after 24 hr. Even at 100 µg/ml, only very sparse gaps are observed. The same trend again is found in the spheroid assay, with statistically significant reduction of spheroid size for both 50 and 100 µg/ml (Figure 4c). Both contraction of the ring and the spheroid, coupled with the migration of the cells through the inner gap of the ring can be seen as the cell's attempt to minimize GNP‐exposed surface area, thereby reducing toxicity.

The survival fraction of LOVO cells following kilo‐voltage irradiation displayed an inverse trend (Figure 5a). The results obtained here were used to guide the selection of radiation dose required for the investigation of the synergistic effect of pGNPs and kilo‐voltage radiation. While it is common to use treatment doses that cause a 50% reduction in survival fraction, this was deemed inappropriate in the current study. At 50% survival from radiation alone, the combination of radiation and pGNPs would leave little room for establishing additional toxicity from any synergy effect that may take place. Therefore, a less cytotoxic dose of 1 Gy was utilized for the combination study, which produced a survival fraction of .79. In combination with pGNPs, this dose was considered most likely to reduce survival fraction to approximately 50%.

The survival fraction of LOVO cells for the same dose was higher when exposed to mega‐voltage radiation than when exposed to kilo‐voltage radiation (Figure 5b). This is expected in view of the higher linear energy transfer of the kilo‐voltage beam relative to the mega‐voltage beam.^30^ In keeping with the aim of achieving a combined 50% reduction in survival, 2 Gy of mega‐voltage radiation was selected, having lowered the survival fraction to .72.

In the pGNP concentration range of 0 to 50 µg/ml, the cells did not exhibit a linear dose response (Figure 5c). Rather, the toxicity appears to be stepwise. The literature on nanoparticle toxicity appears to have conflicting findings. Numerous studies have concluded that no toxicity was observed from their GNP preparation,^25^, ^31^ while others have found otherwise.^24^, ^32^ In our study, we observed a small increase in the survival fraction of cell treated with low concentration of pGNPs, which has not been supported by any previous findings. Further studies are required to explain this phenomenon.

As an initial investigation, the maximum concentration was chosen so as to maximize the potential of the pGNPs to increase the radiation dose while incurring tolerable toxicity. Therefore, 50 µg/ml was selected for further studies.

The statistically significant further reduction in survival fraction in both irradiation energies when treated with pGNP indicates that there is synergy between radiation and pGNPs. While direct comparison of research is difficult, similar levels of enhancement has been achieved previously with mice colorectal adenocarcinoma CT26 cells using smaller GNPs (6.1 nm) and the same radiation energies.^7^ An approximately 20–35% local enhancement of radiation dose potentially translates to a worthwhile contribution to radiotherapy treatment.

Following this initial study, it would be necessary to gain knowledge of the localization and the aggregation behavior of the GNPs in the cells. It is likely that the aggregation characteristics within cells would largely affect both the toxicity and dose enhancement potential of the GNPs. Therefore, this knowledge would be essential in discovering why enhancement and toxicity appear to differ between cells as well as how to maximize the synergy between radiation and GNPs. We are currently performing modeling studies to evaluate the effect of aggregation on dose enhancement.

## 5 Conclusions

We have shown that our PEGylated GNP (pGNP) formulation is nontoxic in LOVO cells up to concentrations of 10 µg/ml. Concentrations of 25 and 50 µg/ml were able to lower cell proliferation in 2D culture. Similar to previous studies, the cells grown in 3D cultures were more resilient, with toxicity observed only at 50 and 100 µg/ml. Interestingly, the cells appear to contract and cluster together in the presence of toxins, which we propose to be a defensive mechanism of the cells to minimize exposure. Ultimately, we demonstrated synergistic effects of pGNPs with both kilo‐voltage and mega‐voltage radiation in LOVO colon cancer cells. We achieved approximately 20–35% increase in cytotoxicity from x‐rays, which suggest great potential for the use of GNPs in radiotherapy.
